# Introital ultrasound in the diagnosis of lower urinary tract symptoms following anti-incontinence surgery using a synthetic midurethral tape

**DOI:** 10.1007/s00192-018-3837-6

**Published:** 2018-12-18

**Authors:** Anna Pawlaczyk, Piotr Wąż, Marcin Matuszewski

**Affiliations:** 1 Department of Urology, Medical University of Gdansk, Gdansk, Poland; 2 Klinika Urologii UCK, ul. Smoluchowskiego 17, 80-214 Gdańsk, Poland; 3 Department of Nuclear Medicine, Medical University of Gdansk, Gdansk, Poland

**Keywords:** LUTS, Midurethral tape, Surgery, Ultrasound, Urinary incontinence

## Abstract

**Introduction and hypothesis:**

Surgical treatment of stress urinary incontinence in women using a synthetic midurethral tape has become a standard procedure. One of the complications observed postoperatively are lower urinary tract symptoms (LUTS). The aim was to analyze the role of introital ultrasound in the identification of patients at risk for developing LUTS after surgical treatment using synthetic tape.

**Methods:**

A group of 50 patients suffering from LUTS following anti-incontinence surgery using synthetic tape was included in this study. The patients with pelvic organ prolapse and coexisting overactive bladder-wet before surgery were excluded. The control group consisted of 50 patients after the same treatment without any complications and with a good outcome. Tape visualization was performed using introital two-dimensional ultrasound. The assessment of the Tape Index (T/U) enabled us to divide the study group into the two subgroups with the tape index value of 0.375 as a borderline. The correlation between the tape position and the occurrence of LUTS was evaluated using a Chi-squared test.

**Results:**

In the group of patients suffering from LUTS, the tape was found to be closer to the bladder neck (the lower edge of the tape was more than 37.5% of the urethral length) and it was statistically significant (Chi-squared = 19.87, *p* < 0.001).

**Conclusions:**

The tape position in the proximal urethra may have an impact on the postoperative occurrence of LUTS. The simple method of introital ultrasound could allow the identification of patients at risk for the development of LUTS after anti–incontinence surgery using synthetic tape.

## Introduction

Stress urinary incontinence is a common medical condition that affects 5–61% of women [1]. For the last 120 years, a lot of effort has been made by surgeons and investigators to create the best model of treatment for stress urinary incontinence. The most widely accepted one has emerged from Petros–Ulmsten’s Integral Theory in 1993. According to this theory, an anatomical defect of the anterior vaginal wall can lead to stress urinary incontinence and overactive bladder. They proposed minimally invasive and simple procedure based on insertion of the polypropylene tape under the midurethra without tension. Therefore, the support for the vaginal hammock will be restored and the activity of the pubo-urethral ligament and levator ani muscles will be reinforced [2].

The technique of the tape insertion is straightforward and relatively reproducible. The success rate is comparable with the former gold standard in anti-incontinence surgery—the Burch procedure [3, 4]. Complications have been analyzed in many separate reports as frequency, nocturia, and voiding dysfunction; reporting frequency in 13% of patients, nocturia in 21%, and voiding dysfunction in 69% [5]. In our study, we defined LUTS as the presence of increased frequency and urgency of urination, nocturia, poor stream, hesitancy, terminal dribbling, and urinary retention. These clinical symptoms may be an early sign of developing bladder emptying and storage disorders, followed by elevated bladder pressure, detrusor overactivity with formation of diverticula, incomplete emptying, increasing chronic urinary retention, recurrent lower urinary tract infections, bladder stone formation, and eventually, upper urinary tract decompensation. The development of the above disorders is long-lasting, and recovery after the removal of the causative factor takes a long time. The most important requirement to avoid it, seems to be a proper localization of the tape, as this is of key significance for appropriate functioning of the lower urinary tract [2, 6, 7]. A tape displacement or incorrect insertion carries the risk of developing complications [5, 7]. In the long-term follow-up after surgery, obstructive voiding, dysuria, overactive bladder and recurrent urinary tract infection may be very significant. The aim of this retrospective case–control study is to find out a simple, practical method of the identification of the patients at risk for developing LUTS after surgical treatment using synthetic tape.

## Materials and methods

Between June 2015 and July 2017, a total of 50 patients were selected and enrolled in the study. They were referred to our center because of symptoms of LUTS following anti-incontinence surgery using polypropylene tape, at various times after the treatment. These complications emerged secondary to anti-incontinence surgery. All 50 patients in the study group were selected on the basis of medical history (medical charts, discharge letters), urogynecological examination, and introital ultrasound with residual urine measurement. There were no patients with coexisting overactive bladder-wet or pelvic organ prolapse before anti-incontinence surgery using polypropylene tape. The patients suffering from pelvic organ prolapse greater than stage I, based on the POP-Q system, were not included. Translated short forms of questionnaires Urogenital Distress Inventory (UDI-6), Incontinence Impact Questionnaire (IIQ-7), and Sandvik, were also completed to assess the patients’ symptoms at the time of inclusion and retrospectively before surgery.

The control group consisted of 50 patients after anti-incontinence surgery using polypropylene tape, treated at our center with a good outcome. Clinical evaluation included medical history, urogynecological examination, and introital ultrasound, with residual volume assessment. The patients also completed questionnaires UDI-6, Sandvik, and IIQ-7 1 month and 6 months postoperatively (Table 1).

**Table 1:** Patient characteristics, average values

Number of patients	LUTS (*n* = 50)	Control group (*n* = 50)
Transobturator tape	24 (48%)	1 (2%)
Retropubic tape	26 (52%)	49 (98%)
Age (on the day of OP), years	59.4 (41–79)	59.0 (42–80)
BMI (on the day of OP), body mass index	28.2 (21–38)	28.6 (20–43)
Time (from the OP to the date of control)	5.3 years (1 month to 19 years)	0.3 year (1–14 months)
Residual volume	82 ml (0–400 ml)	3.42 ml (0–100 ml)
Urogenital Distress Inventory 6	9.2	0.66
Tape index	0.45	0.29
Frequency (0–3)	2.76	0.16
Urgency (0–3)	2.8	0.08

*LUTS* lower urinary tract symptoms (increased frequency and urgency of urination, nocturia, poor stream, hesitancy, terminal dribbling, urinary retention)

Frequency is the frequency of urination scale (0: not at all, 1: slight, 2 moderate, 3: great)

Urgency is the urinary urgency scale (0: not at all, 1: slight, 2: moderate, 3: great)

For the purposes of LUTS evaluation, each patient from the study group and from the control group provided a subjective assessment of the complaints on a scale from 0 to 3 (0: not at all, 1: slight, 2: moderate, 3: great) for frequency (more than 8 micturitions/24 h), urgency of urination, nocturia, poor stream, hesitancy, and terminal dribbling. Urinary retention was measured in milliliters using introital ultrasound - mean values 82 ml (0–400 ml) for the study group and 3.42 ml (0–100 ml) for the control group. Each patient from the study group gave her assessment as 2 or 3 for the most troublesome symptoms—frequency, nocturia, and urgency of urination. In the control group, only one patient gave her assessment as 3 for frequency but 0 for urgency. The mean values for frequency and urgency in the study group were 2.76 and 2.8 respectively and in the control group they were 0.16 and 0.08 respectively. Other LUTS complaints—poor stream, hesitancy, and terminal dribbling—were not so troublesome, and occurred in some patients in the study group but not in the control group.

**Fig. 1:**
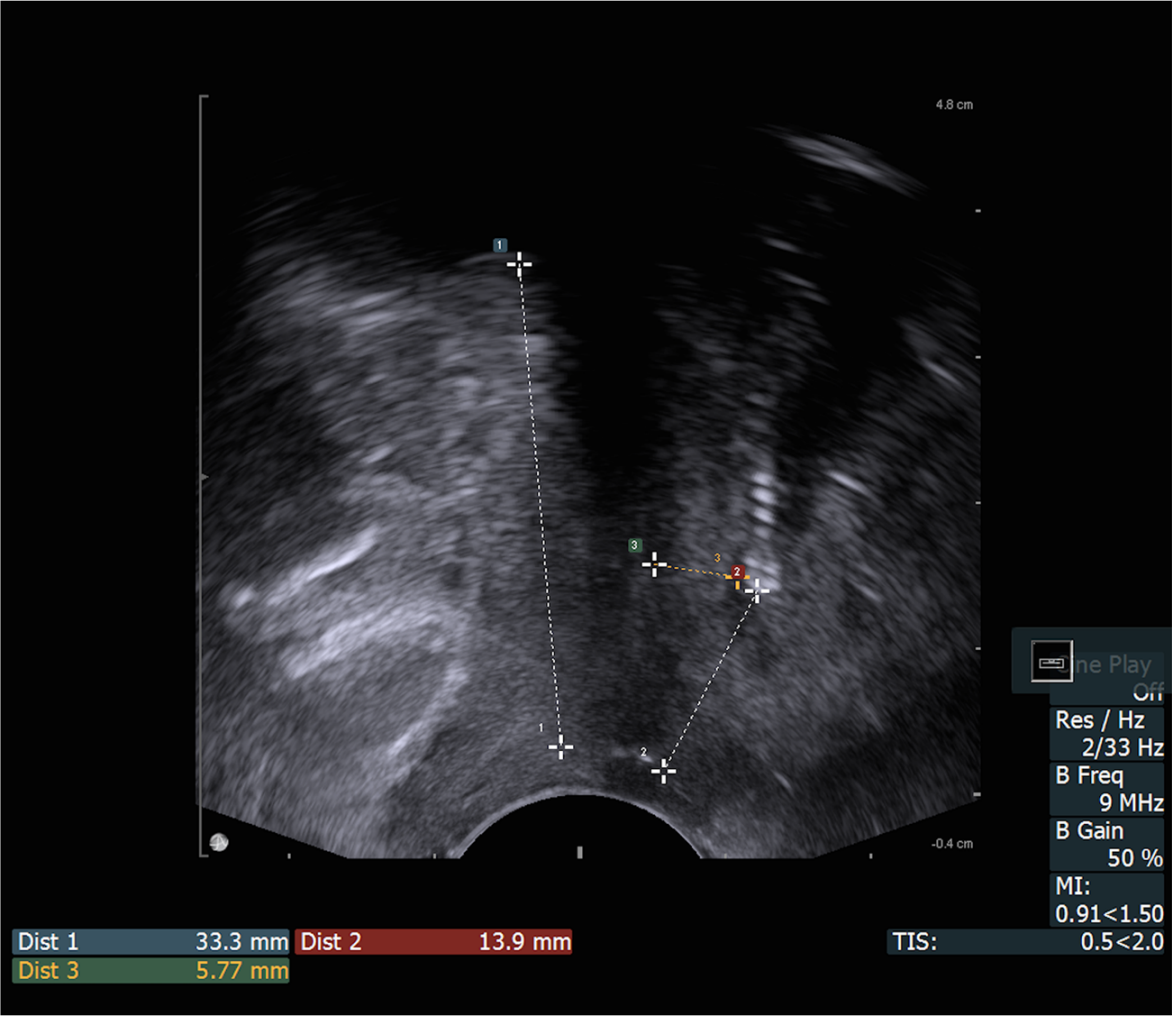
Ultrasound tape visualization in the medio-sagittal plane. *Dist 1*: urethral length. *Dist 2*: distance from the external urethral orifice to the lower edge of the tape. *Dist 3*: distance between the lower edge of the tape and the hypoechogenic urethral complex

The synthetic mesh detection was performed using introital ultrasound. The endocavity probe with a frequency of 9 MHz was used by the same experienced ultrasonographer and the patient remained in the lithotomy position, at rest, after emptying the bladder. Measurements were made only at rest, because many patients were unable to perform the stress test properly and the induced intra-abdominal pressure would not be uniform in all subjects. The surface of the probe was placed in close proximity to the external orifice of the urethra, without pressure on the surrounding structures to avoid anatomical changes. During the ultrasound there were three key measurement points assessed for the study, as depicted on the ultrasound (Fig. 1):

- Urethral length (U)
- Distance between the external urethral orifice and the lower edge of the tape (T)
- Distance between the lower edge of the tape and the hypoechogenic urethral complex (UC, longitudinal smooth muscle and urethral vascularization, T_UC_). The usefulness of tape observation in ultrasound examination has been emphasized by many researchers [5, 7–14]. In each of their works, symphysis pubis was the reference point in the evaluation of the position of the tape. Their measurements of the parameters of the tape position (location and configuration) were assessed in the medio-sagittal plane at rest and on Valsalva. Tape location relative to the urethra was defined by the distance from the bladder neck to the center of the tape. Configuration of the tape was determined as the angle formed by the cranial and caudal aspects of the tape. Our assessment of the tape localization has been based on the most clearly visible points in introital ultrasound—the lower edge of the tape and the external orifice of the urethra.

**Fig. 2:**
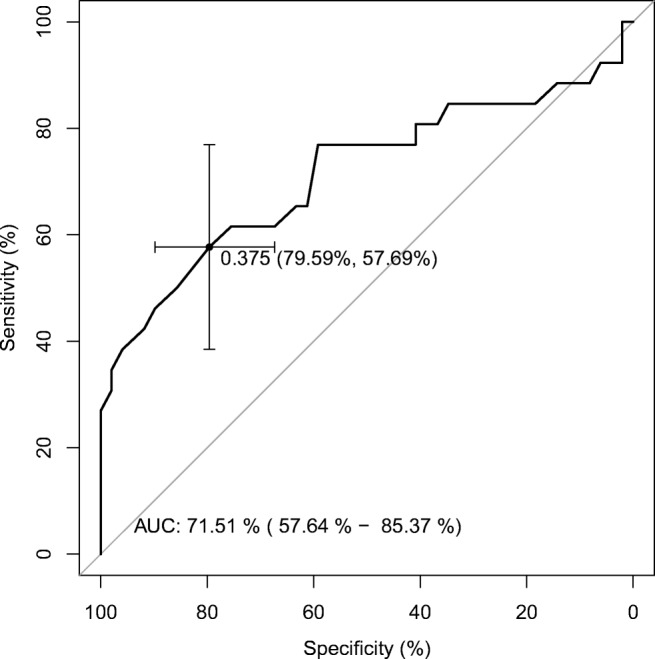
The receiver operating characteristic curve and cut-off point for our model (R software)

In our work, we assessed the value of the tape index, which was determined as a quotient (T/U) showing in a calculable way the position of the tape. The higher it was, the more proximal the tape was situated. In relation to the index, the study group was divided into two subgroups: patients with the tape localized in the distal and medial part of the urethra (the lower edge of the tape was less than 37.5% of the urethral length), and the group of patients with the tape localized in the more proximal part of the urethra (the lower edge of the tape was more than 37.5% of the urethral length). According to the Petros–Ulmsten theory, the tape should be placed without tension under the midurethra; thus, taking into consideration the width of the tape being about 10 mm, it means that the lower edge of the tape should be localized below, in the distal third of the urethra. In our study, we have used 0.375 as the tape index as a borderline identifying the two groups of patients. In the first group, the lower edge of the tape was localized below 0.375 (tape index <0.375). In the second group, the lower edge was localized above 0.375 of urethral length (tape index >0.375). To define the cut-off of T/U we used receiver operating characteristic (ROC) analysis (Fig. 2). We also selected patients with retropubic tape and created the logistic regression model for this subgroup to show that the observed variations are not a function of the tape type. The correlation between LUTS and tape localization determined by the tape index (T/U) was analyzed.

Additionally, the distance from the lower edge of the tape to the hypoechogenic UC in the medio-sagittal view, showing the depth of its position, was measured and the correlation with LUTS was examined by identifying two group of patients:

1. Patients with this distance below 3 mm (T_UC_ < 3 mm)
2. Patients with this distance equal to 1 mm and below (T_UC_ < =1 mm)

The correlation was evaluated using Chi-squared test. *p* < 0.05 was considered statistically significant. This is a retrospective observational case–control study. The study was approved by The Independent Ethics Committee at The Medical University of Gdansk (NKBBN/2/2018).

## Results

A group of 100 patients after anti-incontinence surgery using a polypropylene tape took part in the study: 50 patients suffering from LUTS and 50 patients without those symptoms, as a control group. The shortest period from having the tape placement to inclusion into our study was 1 month and the longest was 19 years (the mean period 5.3 years after surgical treatment). Almost half of the patients (48%) were after the transobturator, and just over half (52%) after retropubic tape placement in the study group.

The mean age and BMI were similar in both groups, and were 59.4 years and 28.2 in the study group and 59.0 years and 28.6 in the control group respectively.

The study group and the control group were different in terms of the technique of the tape placement. In the control group, there were more operated retropubically (98%). The period between surgery and a follow-up (in the control group shorter), the amount of residual volume in the ultrasound assessment, the number of points in UDI-6 (in the study group there was a higher points value for the severity of LUTS), the mean value of the tape index (in the study group the tape was closer to the bladder neck), the severity of the frequency and urgency (in the study group 2.8 on a scale from 0 to 3; Table 1).

The main analysis showed that the localization of the tape in relation to the urethra described by T/U differed significantly in the groups. In the group of patients suffering from LUTS, the tape was found to be closer to the bladder neck (T/U >0.375) and it was statistically significant (Chi-squared = 19.87, *p* < 0.001) (Table 2). Subsequent secondary analysis (the logistic regression model) built for the group of patients with retropubic tape, showed that the increase in the value of T/U by 0.1 gives the odds of belonging of a person with this T/U value to the LUTS+ group 1.97 times larger than belonging to the LUTS- group.

**Table 2:** Association between ultrasound parameters and LUTS

	LUTS (+)	LUTS (−)	Chi-squared	*p* value	Odds ratio	Confidence interval
T/U < 0.375	17	38	19.87	*p* < 0.001	6.15	(1.95, 19.38)
T/U > 0.375	33	12				
T-UC <3	17	10	2.49	NS 0.10 < *p* < 0.20	2.06	(0.63, 6.79)
T-UC > =3	33	40				

Level of significance being taken as *p* < 0.05. Confidence level: 99%

With our initial assessment, we did not find any correlation between the distance from the tape to the urethra (UC) and the occurrence of LUTS in the group of patients if the distance was less than 3 mm (Chi-squared = 2.49, Chi-squared _obs_ < Chi-squared _0.1_).

However, our subsequent secondary analyses showed that, if the distance was equal to 1 mm or less, meaning that the tape was situated very close to the urethra, the correlation with the occurrence of LUTS was observed and this was statistically significant (Chi-squared = 5.32, *p* < 0.001).

## Discussion

Surgical treatment of stress urinary incontinence in women using the midurethral polypropylene tape is nowadays recognized as the best treatment method. Every year, thousands of these procedures are performed. According to the authors of the method, a very important condition for a successful result is the long-lasting location of the tape precisely under the midurethra. Inappropriate insertion or subsequent tape migration toward the bladder can be related to failure: persistent or recurrent stress urinary incontinence or the occurrence of LUTS, which is much more bothersome for the patients than the recurrence of stress urinary incontinence.

Several studies report the occurrence of LUTS among 1.6–69% patients after anti-incontinence surgery using midurethral tape, which is considered treatment failure [5, 8, 9]. Urologists and urogynecologists often consider these cases as de novo overactive bladder, indicating the tight location of the tape in relation to the urethra. In our study, the patients often postponed the consultation with the doctor for several years, accepting LUTS as the normal outcome after anti-incontinence surgery. Leaving a patient with LUTS without treatment may lead to serious diseases in the lower urinary tract (detrusor overactivity, bladder diverticula, recurrent urinary tract infection (UTI), bladder stone formation) and later, the upper part of the urinary tract (reflux, hydronephrosis, kidney function impairment) could also be affected. The concept of wrong placement or migration of the tape will give a new perspective on this problem, especially when we have available a straightforward and reproducible method of evaluation.

The examination of the lower urinary tract using the introital ultrasound allows us to see the proper place for the tape insertion and to check postoperatively its correct functioning. The high-value visualization using this method in the evaluation after anti-incontinence surgery was declared in several reports [5, 7, 10–13]. Among patients suffering from LUTS after anti-incontinence surgery, we observed the localization of the tape under the proximal part of the urethra more often and it was statistically significant.

The other parameter describing the tape location, which has a minor impact on the occurrence of LUTS, is the distance from the tape to the hypoechogenic urethral complex measured in the sagittal plane on the level of the lower edge of the tape. Rautenberg et al. postulated that this distance, which is less than 3 mm, would be of clinical significance, but in his work a distance was measured between the tape and UC in the sagittal plane at the central level of the implant [9]. We observed the lower edge of the tape to be the most important point of the action during a stress test performed under postoperative ultrasound evaluation. In our study, we found that when the distance from the lower edge of the implant to the UC was shorter than 1 mm, it had a negative impact on the surgical outcome.

The method of depicting the position of the tape used in our study is straightforward and allows us in a quick and uncomplicated way to draw conclusions of clinical importance. The patient does not require any special pre-preparation. Described by other authors, bladder filling of 300 ml to perform the ultrasound investigation was abandoned in our study because of the lack of compliance among patients suffering from LUTS. We assessed neither angles nor gaps between the tape and symphysis pubis during Valsalva and cough, as we found a lack of reproducibility among our study population [5, 12, 14].

Dietz et al. assessed the craniocaudal distance between the upper edge of the tape and the inferoposterior margin of the symphysis pubis using a sector abdominal probe for translabial ultrasound [5]. Rautenberg calculated the central point of the tape, from which he set up all ultrasound parameters [9]. During our observations of the dynamic ultrasound tests, we saw the lower edge of the tape as being the most important. For this reason, we determined this point in assessing tape localization, calculating the Tape Index. We have abandoned the use of bony pelvis or the bladder neck as markers for assessing ultrasound parameters in the localization of the tape because of the lack of anatomical uniformity [15]. The distance measured from the bladder neck to the tape depends on pelvic floor muscle activation and therefore it was not satisfactory.

Based on our observations and reports from the literature, we suggest that introital ultrasound could be the method of choice for the evaluation of the mislocation and dysfunction of polypropylene implants. The relationship between the position of the tape under the proximal part of the urethra and treatment failure suggested by others was also confirmed in our study [5, 7, 12].

It is worth noting that the lack of guidelines in preoperative and postoperative assessment makes it difficult to compare the results of treatment with the polypropylene tape. The method of ultrasound assessment presented in our study enables a very reliable, inexpensive, simple, and non-invasive method of control of the tape position among all patients after surgery.

This study comprises a heterogeneous population of a study group and a control group. All patients in the control group were treated in our department, whereas in the study group, only 18 of them were (36%). In the control group, the time of observation after surgery was much shorter than in the study group and we are aware that there is a possibility of developing complications after a longer period of observation. Despite the aforementioned reservations, the conclusions regarding the relationship between the location of the tape and the occurrence of LUTS are consistent and prove the relevance of the appropriate location of the implant. It should also be noted that patients with LUTS were not examined immediately after surgery, and that their medical history was analyzed according to the medical charts and retrospectively collected questionnaires. The beginning of LUTS is difficult to determine. Some patients accepted LUTS as a normal outcome after anti–incontinence surgery. The emergence of these types of complaints in connection with the insertion of a polypropylene tape under the midurethra can be an early sign of developing bladder emptying and storage disorders, which could lead to the development of irreversible lesions in the lower urinary tract (detrusor overactivity, bladder diverticula, recurrent UTI, bladder stone formation).

## Conclusion

Migration of the polypropylene tape in the cephalic direction, or putting it too proximal (lower edge of the tape above 0.375) may result in the development of troublesome complaints from the lower urinary tract defined as LUTS. Patients with severe LUTS, despite being cured of stress urinary incontinence, assessed quality of life as being worse than before the operation. It was also confirmed that the immediate proximity of polypropylene material (1 mm or less) from the UC may be associated with the development of LUTS. The introduction of introital ultrasound as a routine tool in postoperative control after implantation of the tape could allow the identification of patients at risk for developing LUTS or qualification for re-operation in those who experienced the symptoms after the operation with use of the tape.
